# Beyond Mallampati: From Universal Bedside Tests to Precision Airway Risk Stratification

**DOI:** 10.3390/jpm16100532

**Published:** 2026-10-16

**Authors:** Daniele Salvatore Paternò, Luigi La Via, Flavia Paola Arena, Francesca Barbagallo, Basilio Davide Lesina Calà, Emilia Concetta Lo Giudice, Rita Cataldo, Massimiliano Sorbello

**Affiliations:** 1 Department of Anaesthesia and Intensive Care, “Giovanni Paolo II” Hospital, 97100 Ragusa, Italy; massimiliano.sorbello@unikore.it; 2 Department of General Surgery and Medical Surgical Specialties, University of Catania, 95123 Catania, Italy; luigi.lavia@unict.it; 3 Postgraduate School of Anaesthesia and Intensive Care, “Kore” University, 94100 Enna, Italy; flaviapaola.arena@unikorestudent.it (F.P.A.); francesca.barbagallo002@unikorestudent.it (F.B.); basiliodavide.calalesina@unikorestudent.it (B.D.L.C.); 4 Department of Anaesthesia and Intensive Care, “Umberto I” Hospital, 94100 Enna, Italy; emilia.logiudice@unikore.it; 5 School of Medicine, Anaesthesia and Intensive Care, “Kore” University, 94100 Enna, Italy; 6 Unit of Anaesthesia and Intensive Care, Fondazione Policlinico Universitario Campus Bio-Medico, 00128 Rome, Italy; r.cataldo@unicampus.it; 7 Research Unit of Anaesthesia and Intensive Care, Department of Medicine and Surgery, Università Campus Bio-Medico, 00128 Rome, Italy

**Keywords:** airway management, difficult intubation, precision medicine, personalised medicine, point-of-care ultrasound, machine learning, risk stratification, perioperative care, videolaryngoscopy, patient phenotype

## Abstract

Airway management remains one of the few domains of anaesthetic practice still governed by universal, dichotomous screening thresholds applied to profoundly heterogeneous populations. Conventional bedside tests—the modified Mallampati test, thyromental distance and the upper lip bite test—retain modest and inconsistent diagnostic accuracy, leaving the majority of difficult intubations unanticipated while generating false alarms that drive resource-intensive contingency plans. This narrative review reframes preoperative airway evaluation as a problem of individualised risk stratification rather than population screening. We first summarise the performance limits of classical bedside predictors and the reasons for their ceiling effect, including low target prevalence, inter-observer variability, spectrum bias and heterogeneous outcome definitions. We then examine the data streams reshaping the field: point-of-care upper airway ultrasound, whose parameters provide operator-accessible and objective anatomical information; cross-sectional and craniofacial imaging; and machine-learning models based on clinical variables, facial images and voice analysis. For each we discuss accuracy, reproducibility, validation status and barriers to adoption. We then propose a phenotype-oriented framework in which assessment intensity is matched to pre-test probability, with dedicated pathways for obesity and obstructive sleep apnoea, obstetric, head-and-neck oncological and post-radiotherapy, critically ill and emergency, and paediatric populations. We map this framework onto the 2025 European Society of Anaesthesiology and Intensive Care and Difficult Airway Society recommendations and outline the validation, calibration and equity requirements—including algorithmic bias across skin tone, sex and ethnicity—that must be met before precision airway assessment can enter routine practice.

## 1. Introduction

Securing the airway is among the most routine, the most consequential and the most poorly predicted interventions in medicine. The Fourth National Audit Project (NAP4) of the Royal College of Anaesthetists and the Difficult Airway Society (DAS) estimated 46 major airway complications per million general anaesthetics—about one in 22,000—with a mortality of 5.6 per million; airway management was judged good in only 19% of assessable cases, and statistical modelling suggested that as few as 25% of relevant incidents may have been reported [1]. Outside the operating theatre, the picture is worse: in intensive care units (ICUs) and emergency departments (EDs), 61% and 31% respectively of reported airway events led to death or persistent neurological injury, and failure to use or correctly interpret capnography contributed to 74% of these outcomes [2].

**Figure 1:**
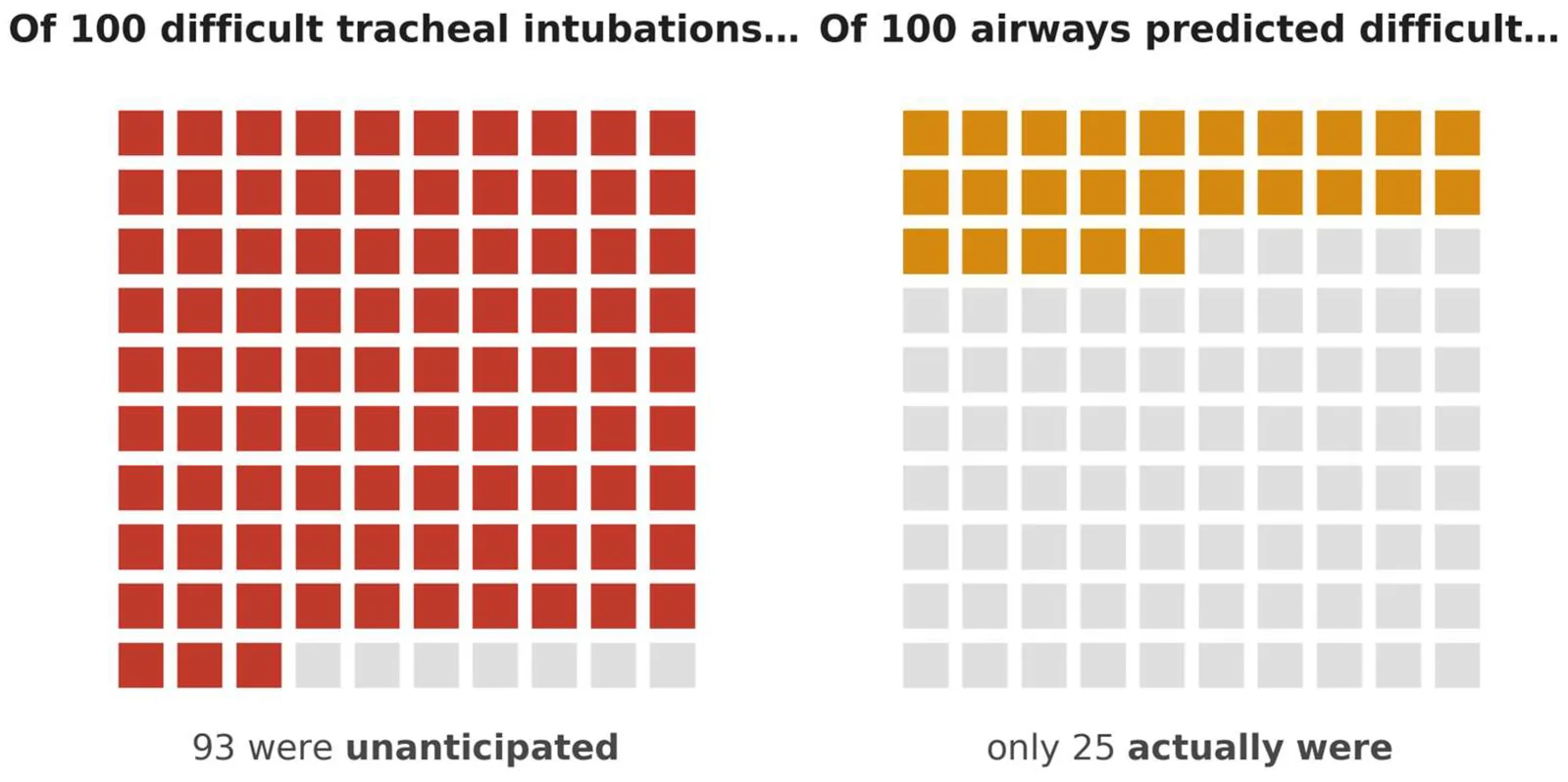
The prediction paradox in airway management. Data from a cohort of 188,064 patients registered in the Danish Anaesthesia Database [3]. (**Left**): of every 100 difficult tracheal intubations encountered, 93 occurred in patients in whom difficulty had not been anticipated. (**Right**): of every 100 patients in whom difficulty was anticipated, only 25 proved genuinely difficult. The corresponding proportions for difficult facemask ventilation were 94% and 22%, respectively. Preoperative airway assessment as currently practised therefore fails in both directions simultaneously: it misses the large majority of the events it exists to detect, while the alarms it does raise are wrong about three times out of four.

Most of these events are not foreseen. In a cohort of 188,064 patients from the Danish Anaesthesia Database, 3154 of 3391 difficult intubations (93%) were unanticipated; conversely, when difficulty was anticipated, only 229 of 929 patients (25%) proved genuinely difficult. For difficult mask ventilation the figures were 94% and 22% respectively [3]. In other words, contemporary preoperative airway assessment misses most of the events it is designed to detect while raising an alarm that is wrong three times out of four (Figure 1). This persistent unreliability has been described as anaesthesia’s Achilles’ heel [4].

The specialty has responded mainly by improving rescue rather than prediction. The 2025 Difficult Airway Society guidelines for the management of unanticipated difficult tracheal intubation in adults are an excellent example: they refine what to do once difficulty declares itself, on the reasonable assumption that it will often not be predicted [5]. This is sound risk management, but it does not address the upstream problem. The 2025 update of the European Society of Anaesthesiology and Intensive Care (ESAIC) guidelines on preoperative assessment goes further, recommending that multiple tests be combined to improve predictive values (grade 1A), that the minimum set of tests vary between patients according to the underlying pathology (grade 2C), and that it extend beyond anatomy to encompass physiology, environment, devices and individual and team expertise (grade 1C) [6]. The direction of travel is therefore already established at guideline level; what is missing is an account of how these principles translate into a concrete assessment strategy for identifiable patient groups. Existing reviews of airway assessment have catalogued the available tools [7], and we have ourselves recently reviewed two of the modalities that are reshaping the field, artificial intelligence (AI) [8] and point-of-care ultrasound (POCUS) [9]. Yet across this literature the organising logic has remained that of a universal screening programme: one battery of tests, one set of thresholds, applied identically to a 25-year-old undergoing knee arthroscopy and a 70-year-old with a post-radiotherapy neck. The present review differs in scope and in intent from those earlier appraisals: rather than evaluating a single modality, it asks how the available modalities should be combined and, above all, to whom each should be applied.

Precision medicine offers a different organising logic: diagnostic and therapeutic intensity should be matched to individual risk, estimated from the richest available combination of data, rather than applied uniformly. Applied to the airway, this reframing has three practical consequences. The relevant question ceases to be “is this test positive?” and becomes “what is this patient’s pre-test probability, and what additional information would change management?”. “Difficulty” must be recognised as a property of the encounter between patient, operator and environment, not of the patient alone. And assessment resources—ultrasound, imaging, senior review, awake techniques—should be concentrated where the expected yield is greatest rather than distributed evenly.

This review is therefore not a further catalogue of airway tests. Its aims are as follows: (i) to establish why universal bedside screening has reached a performance ceiling; (ii) to appraise the multimodal data streams—point-of-care ultrasound, imaging and machine learning—that can inform an individualised risk estimate; (iii) to propose a phenotype-oriented framework in which assessment intensity is proportional to pre-test probability, mapped onto contemporary guidelines; and (iv) to define the validation, calibration and equity conditions required before such an approach enters routine practice.

## 2. Materials and Methods

This is a narrative review, prepared with attention to the six domains of the Scale for the Assessment of Narrative Review Articles (SANRA) [10]. PubMed/MEDLINE was searched from inception to 31 July 2026, combining controlled vocabulary and free-text terms for airway management, difficult intubation, difficult laryngoscopy, difficult mask ventilation, airway assessment, point-of-care ultrasound, imaging, machine learning and artificial intelligence, together with population-specific terms (obesity, obstructive sleep apnoea, obstetric, head and neck neoplasms, critical illness, paediatrics) and methodological terms (guidelines, external validation, algorithmic fairness). The search was supplemented by hand-searching the reference lists of all retrieved systematic reviews, meta-analyses and society guidelines. The complete search blocks, selection criteria and prioritisation rules are provided in Supplementary Material S1.

Priority was given to systematic reviews, meta-analyses, large prospective cohorts, registries, randomised trials and society guidelines published in English; primary observational studies were included where they illustrated a parameter, a population or a methodological limitation not otherwise represented. No dual independent screening, formal quality scoring or quantitative synthesis was undertaken, and study selection reflects the authors’ judgement of representativeness. Comparative expressions used below, such as “best-supported” or “most extensively studied”, therefore refer to the body of evidence retrieved by this search and summarised in Table 1 and Table 2, and are not the result of a formal ranking; where estimates derive from different evidence syntheses, this is stated explicitly. These are acknowledged limitations of the narrative format.

## 3. The Ceiling of One-Size-Fits-All Bedside Screening

### 3.1. What the Evidence Actually Shows

The diagnostic accuracy of bedside airway tests has been quantified with unusual rigour, and the conclusions are consistent across independent syntheses. The Cochrane review of airway physical examination tests in apparently normal adults found that all index tests had relatively low sensitivities with high between-study variability, while specificities were consistently and markedly higher; the modified Mallampati test (MMT) had the highest sensitivity for difficult tracheal intubation among the tests examined, and the upper lip bite test (ULBT) showed the most favourable overall properties. For difficult mask ventilation, the MMT achieved a summary sensitivity of only 0.17 (95% CI 0.06–0.39) against a specificity of 0.90 (95% CI 0.81–0.95) [11]. A subsequent meta-analysis of 227 articles encompassing 686,089 patients reported pooled sensitivity/specificity of 0.39/0.86 for the MMT, 0.38/0.83 for thyromental distance and 0.52/0.84 for the ULBT [12].

The Rational Clinical Examination systematic review, pooling 62 high-quality studies and 33,559 patients, estimated the prevalence of difficult intubation at 10% (95% CI 8.2–12%) and identified the findings with genuine discriminatory value: a class 3 ULBT (positive likelihood ratio 14, 95% CI 8.9–22; specificity 0.96) and a short hyomental distance (positive likelihood ratio 6.4, 95% CI 4.1–10; specificity 0.97) [13]. The pattern is consistent. These tests are highly specific and, when positive, meaningfully raise the probability of difficulty; they are poorly sensitive, and their absence is close to uninformative. They behave, in short, as confirmatory tests being deployed as screening tests.

The response of the literature to this ceiling has been to propose new bedside measurements, and their trajectory is instructive. A systematic review of 27 studies and 18,141 patients confirmed the favourable specificity of the ULBT—exceeding 85% in all but one study—but found sensitivity above 70% in only 11 of 27 studies [14]. Thyromental height has been the most promising recent candidate, with two meta-analyses reporting summary sensitivity of 82.6% and specificity of 93.5% (AUC 81.1%) [15] and an AUC of 85% (95% CI 81–88%) [16] respectively; both, however, explicitly caution that high risk of bias across the primary studies and unstandardised external laryngeal manipulation may have inflated these estimates, and neither has been followed by external validation or by demonstrated impact on outcome. Anthropometric measures fare less well: in a head-to-head comparison of five tests, neck circumference was statistically indistinguishable from thyromental distance and the MMT, and inferior to both the ULBT and the ratio of height to thyromental distance [17]. Two decades of new bedside indices have therefore produced variations around the same operating point rather than a shift away from it.

For difficult mask ventilation the picture is comparable but the risk factors differ in emphasis: a meta-analysis of 20 observational studies (335,846 patients) reported a prevalence of 6.1% overall and 14.4% in patients with obesity, with the strongest associations for previous neck radiotherapy (OR 5.0), increased neck circumference (OR 4.04), obstructive sleep apnoea (OSA) (OR 3.61), the presence of a beard (OR 3.35), snoring (OR 3.06), obesity (OR 2.99) and male sex (OR 2.76)—with Mallampati III–IV only eighth in rank (OR 2.36) [18]. Bedside tests optimised for laryngoscopy are therefore not automatically transferable to the ventilation domain, a distinction routinely blurred in practice.

### 3.2. Why the Ceiling Exists

Four mechanisms explain why refining individual tests has yielded diminishing returns.

This is not a novel observation but an increasingly explicit one: the ESAIC guidelines state that the simple application of statistics to single or multiple tests yields a high number of false positives and a dangerously low number of false negatives, and that a purely statistical approach to a static anatomical examination “will always result in poor predictive values” [6].

Low prevalence and the arithmetic of positive predictive value. With a pooled prevalence of difficult intubation of approximately 10% in surgical series [13]—and considerably lower in unselected routine practice—and specificities in the 0.83–0.90 range [12], even a highly sensitive test generates more false than true positives. This is not a remediable flaw of any particular test; it is a property of screening for uncommon events, and it explains directly why anaesthetists’ own predictions carry a positive predictive value of only 25% [3].

Inter-observer variability. The MMT and the ULBT require standardised patient positioning, phonation and effort, and agreement between assessors is imperfect. When two blinded assessors examined the same 101 adults using 15 predictive tests, agreement was only satisfactory for the MMT (κ = 0.50) and fair for the ULBT (κ = 0.37), while thyromental distance and cervical spine mobility—both measured, not graded—showed essentially no agreement (intraclass correlation coefficients of −0.002 and 0.02 to 0.14 respectively); the authors concluded that poor reproducibility may itself be one reason why prediction models are not universally reliable [19]. An earlier four-observer study reached the same conclusion for thyromental distance and neck mobility, while finding acceptable agreement for mouth opening, jaw protrusion and the MMT [20], and reliability studies of the ULBT and MMT confirm meaningful discordance [21]. A test that two competent clinicians score differently cannot support a threshold applied uniformly across a population.

Spectrum bias and outcome heterogeneity. Most accuracy studies exclude patients with overt airway pathology—precisely the population in whom prediction matters most—while the reference standard varies between Cormack–Lehane grade ≥ 3, the Intubation Difficulty Scale, number of attempts and operator gestalt. Pooled estimates therefore aggregate outcomes that are not the same event. This is beginning to be addressed at source. The recently developed MASCAN score provides an objective, data-driven classification of difficult facemask ventilation built from five observable indicators—two-handed grip, oral airway use, jaw thrust, tidal volume ≤ 2 mL·kg^−1^ and a fall in peripheral oxygen saturation ≥ 10%—reaching an area under the receiver operating characteristic curve of 0.94 for the score and 0.96 for the underlying model in a prospective cohort of 400 patients, in whom difficult facemask ventilation occurred in 10.8% [22]. Agreeing what counts as “difficult” is a precondition for comparing predictors at all, and its absence has quietly limited every synthesis discussed above.

The operator and the environment are unmeasured covariates. No bedside test incorporates who will intubate, with what device, at what hour, with what assistance. Yet these variables demonstrably influence outcome, as the divergence between operating-theatre and out-of-hours ICU/ED event severity illustrates [2].

### 3.3. Composite Scores: A Partial Answer

Multivariate indices were the specialty’s first attempt at individualisation, and they do outperform single tests. In a prospective comparison of three models, areas under the curve were 0.834 (Langeron), 0.805 (Naguib) and 0.752 (El-Ganzouri), with sensitivities of 85.7%, 67.9% and 35.7% respectively [23]; extending the El-Ganzouri index with neck circumference and body mass index (the EL.GA+ score) improved predictive value in patients with mild obesity [24]. In the ICU, the MACOCHA score—combining patient factors (Mallampati III–IV, OSA, reduced cervical mobility, limited mouth opening), pathology (severe hypoxaemia, coma) and operator (non-anaesthetist)—achieved an AUC of 0.89 in development and 0.86 in external validation, with sensitivity 73%, specificity 89% and a negative predictive value of 98% against a positive predictive value of 36% [25].

MACOCHA is the clearest existing proof of concept for this review’s argument: its performance derives precisely from combining anatomical, physiological and contextual data, and from being developed in a defined population rather than in “all adults”. Yet the limits of scores alone were exposed by the DIFFICAIR cluster-randomised trial, in which mandatory departmental implementation of the Simplified Airway Risk Index did not reduce unanticipated difficult intubation compared with usual care (2.38% vs. 2.39%; adjusted OR 1.03, 95% CI 0.77–1.38; *p* = 0.84) [26]. Better arithmetic applied to the same limited inputs does not solve the problem; richer inputs may. Table 1 summarises the pooled performance of the principal bedside tests and composite indices.

**Table 1:** Diagnostic performance of conventional bedside airway tests (pooled estimates).

Test	Pooled Sensitivity	Pooled Specificity	Best-Evidence LR+	Principal Limitation
Modified Mallampati test	0.39 [12]	0.86 [12]	Modest	Inter-observer variability; sensitivity 0.17 for difficult mask ventilation [11]
Thyromental distance	0.38 [12]	0.83 [12]	Modest	Threshold heterogeneity (<6–<7 cm)
Upper lip bite test	0.52 [12]	0.84 [12]	14 (8.9–22) for class 3 [13]	Highly specific but insensitive; unusable in edentulous patients
Short hyomental distance	Low	0.97 [13]	6.4 (4.1–10) [13]	Confirmatory rather than screening role
Mouth opening	Low	High [11]	Modest	Poor stand-alone discrimination
Composite indices (Langeron, Naguib, El-Ganzouri)	35.7–85.7% [23]	Variable	AUC 0.75–0.83 [23]	No outcome benefit when implemented departmentally [26]
MACOCHA (ICU)	73% [25]	89% [25]	AUC 0.86–0.89 [25]	Population-specific; PPV 36%

## 4. Multimodal Data Streams for Individualised Assessment

### 4.1. Point-of-Care Upper Airway Ultrasound

Ultrasound moves airway assessment from surface inspection to measurement of the tissue that obstructs the line of sight. Anterior neck soft-tissue thickness at defined landmarks quantifies what Mallampati grading only infers.

The evidence base has matured rapidly. A meta-analysis of 15 studies reported, for prediction of difficult direct laryngoscopy, sensitivity/specificity of 0.82/0.79 for distance from skin to epiglottis (DSE) with an AUC of 0.87 (95% CI 0.84–0.90), against AUCs of 0.77 for distance from skin to hyoid bone and 0.78 for distance from skin to vocal cords [27]. The most recent and largest synthesis—60 studies, 10,580 patients, 58 parameters—found for difficult laryngoscopy a sensitivity of 0.84 (95% CI 0.74–0.91) and specificity of 0.81 (95% CI 0.61–0.92) for the skin-to-vocal-cords distance (AUROC 0.87; high certainty of evidence), and for difficult tracheal intubation a sensitivity of 0.80 (95% CI 0.74–0.85) and specificity of 0.86 (95% CI 0.74–0.91) for the skin-to-epiglottis distance (high certainty), with the skin-to-hyoid distance achieving the highest AUROC at 0.86 [28]. Independent systematic reviews converge on the same three parameters plus the hyomental distance ratio, and report that combinations of clinical and sonographic parameters outperform either alone [29,30,31,32]; primary prospective studies across differing healthcare settings—elective surgical, emergency and mixed adult populations—reproduce the pattern with reasonable consistency [33,34,35], and ultrasound has been proposed as a combined assessment and procedural tool in the emergency airway [36].

Three implications follow, the first requiring care in its statement. Pooled sensitivity for the skin-to-epiglottis distance (0.80–0.82) is approximately twice that reported for the MMT (0.39), but the estimates come from separate evidence syntheses with different populations and reference standards rather than from head-to-head comparison, so the difference indicates the possible magnitude of a gain rather than a measured effect size. Second, ultrasound partially escapes the inter-observer problem, since it produces a continuous measurement rather than a graded visual impression, although absolute cut-offs remain population-dependent and require local calibration [37,38]. Third, and most relevant to a precision framework, ultrasound parameters retain performance in the populations where bedside tests fail most conspicuously: pregnant patients [39], patients with obesity [40] and children, in whom the hyomental distance ratio achieved an area under the curve of 0.959 for difficult laryngoscopy in those under two years of age [41].

Ultrasound also has a role beyond prediction. Digital palpation identified the cricothyroid membrane (CTM) in only 39% of obese women in labour compared with 71% of non-obese women, with the error increasing with neck circumference [42]: in exactly the patients most likely to need front-of-neck rescue, the landmark is least likely to be found without ultrasound. Pre-procedural CTM marking is a concrete, low-cost expression of precision airway management.

**Figure 2:**
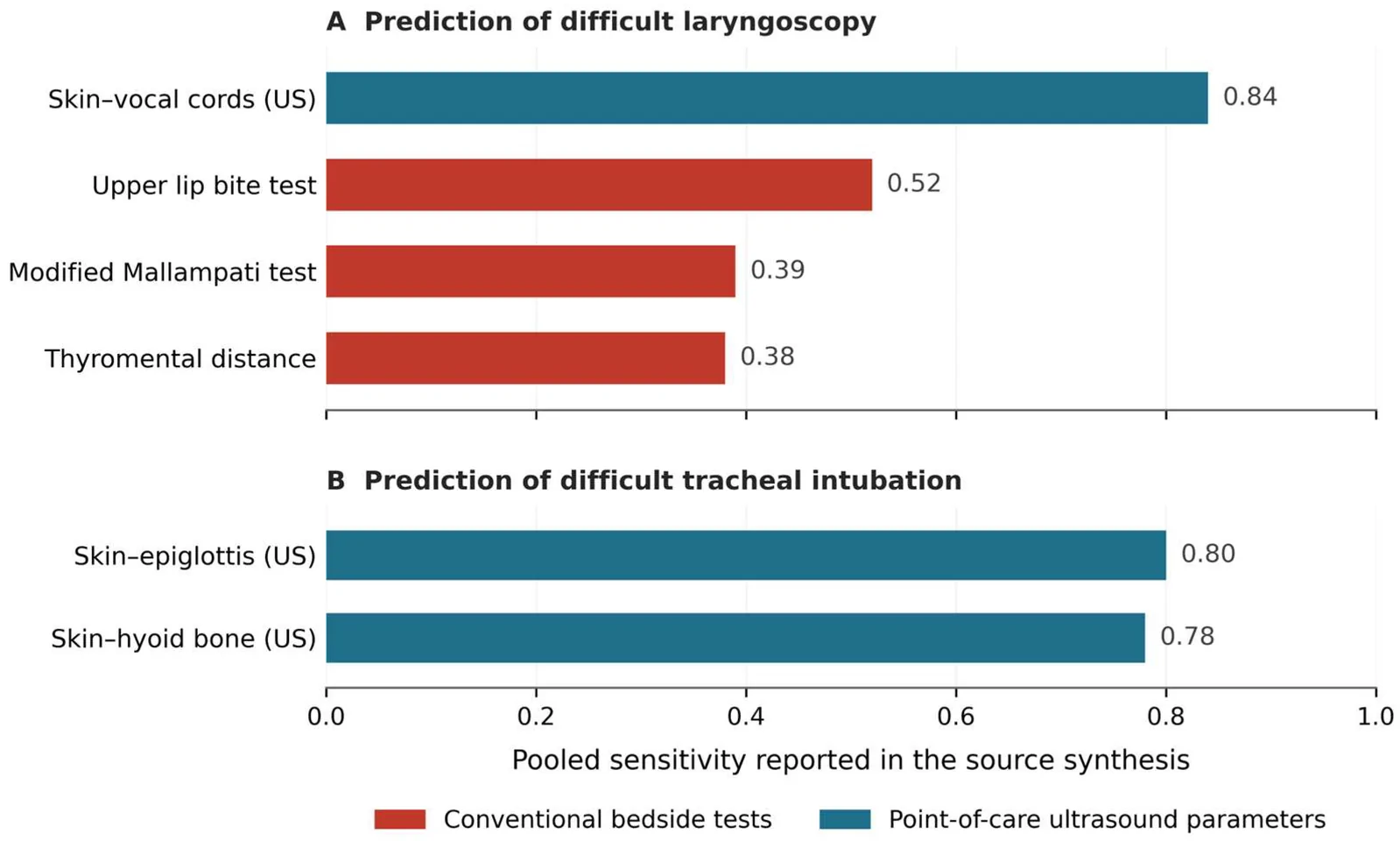
Pooled sensitivity reported for conventional bedside tests and for point-of-care ultrasound parameters, separated by target outcome. (**A**) Prediction of difficult laryngoscopy. (**B**) Prediction of difficult tracheal intubation. Data from [12,27,28]. The two outcomes are shown in separate panels because they are related but distinct events, and the estimates within each panel are drawn from different evidence syntheses using different populations and reference standards rather than from head-to-head comparison; the figure is therefore an indirect illustration of the pattern discussed in Section 4.1 and does not constitute a formal comparison of tests. No threshold is shown, since no consensus minimum sensitivity for preoperative airway screening tests has been established. Specificity, on which the bedside tests perform considerably better, is reported in Table 1 and Table 2.

The clinical translation, stated cautiously, is that a negative bedside examination provides weak reassurance, and that sonographic measurement appears to provide more—though how much more, and whether acting on it changes outcome, remains to be established (Figure 2).

The sonoanatomy of the upper airway, the technique of each measurement and the additional intraprocedural applications of ultrasound—tracheal tube confirmation, guidance of front-of-neck access, and the emerging role of AI in image interpretation—have been detailed elsewhere and are not reproduced here [9]; the point relevant here is narrower—ultrasound supplies a continuous, patient-specific anatomical variable of a kind that no bedside test provides.

One qualification is essential. Notwithstanding the diagnostic accuracy summarised above, the 2025 ESAIC guidelines concluded that the evidence remains insufficient to recommend instrumental tests such as ultrasound for routine airway assessment, issuing this only as a clinical practice statement [6]. The discrepancy is instructive rather than contradictory: the available studies establish that sonographic parameters discriminate, but not that measuring them changes management or outcome. This is precisely the distinction between diagnostic accuracy and clinical utility developed in Section 7, and it defines the evidence that the field still owes itself.

Two obstacles therefore remain, and they are of different weight. The evidential one is that identified above and by ESAIC: discrimination has been demonstrated, clinical utility has not, and only prospective studies in which management is actually altered by the measurement can close that gap. The practical ones—training and credentialing, examination time in a high-throughput list, probe availability, absent standardised reporting—are those that lung and cardiac point-of-care ultrasound overcame within a decade, and are the easier of the two to solve. The principal sonographic parameters, their target outcomes and their reported accuracy are summarised in Table 2.

**Table 2:** Principal sonographic parameters for airway assessment.

Parameter	Target Outcome	Reported Accuracy	Comment
Distance from skin to epiglottis (DSE)	Difficult laryngoscopy/intubation	Sens 0.80–0.82; spec 0.79–0.86; AUC 0.87 [27,28]	Best-supported single parameter; midway, neutral head position
Distance from skin to hyoid bone	Difficult intubation	Sens 0.78; spec 0.81; AUROC 0.86 [28]	Highest AUROC in the largest synthesis
Distance from skin to vocal cords	Difficult laryngoscopy	Sens 0.84; spec 0.81; AUROC 0.87 [28]	High certainty of evidence
Hyomental distance ratio (extended/neutral)	Difficult laryngoscopy	Consistently associated across reviews [29,37,43]	Dynamic measure; captures cervical extension
Pre-epiglottic space/epiglottis–vocal cord ratio	Difficult laryngoscopy	AUC 0.71 [27]	Weaker; of mechanistic interest
Tongue thickness	Difficult facemask ventilation	Narrative association only [28]	The only ultrasound signal for the ventilation domain
Cricothyroid membrane identification	Front-of-neck access	Palpation accurate in 39% of obese vs. 71% of non-obese parturients [42]	Pre-procedural marking in high-risk patients

### 4.2. Cross-Sectional and Craniofacial Imaging

A substantial minority of patients arrive in the anaesthetic room with imaging that already contains the answer. Computed tomography and magnetic resonance imaging performed for oncological staging, cervical spine assessment or trauma depict precisely the structures that determine laryngoscopic view, yet are seldom interrogated for airway purposes. In a randomised controlled study, three-dimensional computed tomography reconstruction with virtual endoscopy significantly changed the anaesthetist’s choice of tracheal tube size and informed nostril selection for nasotracheal intubation, without altering the number of attempts or complications [44]: it changed the plan rather than the outcome, which is what an assessment tool should be expected to do first. Simple radiographic indices retain value in defined populations: in cervical spondylosis, a radiographic cervical curvature angle ratio achieved the largest area under the curve (0.714, 95% CI 0.633–0.794) among mobility indicators for difficult laryngoscopy [45].

This is a low-cost opportunity with a clear precision logic: rather than acquiring new tests in unselected patients, extract existing high-resolution data in patients who are already known to be high-risk. The principal obstacles are workflow (radiology reports are not written to answer airway questions) and the absence of validated, reproducible airway metrics on cross-sectional imaging.

### 4.3. Machine Learning and Artificial Intelligence

AI enters this field with a specific and legitimate claim: to integrate more variables, and subtler ones, than a human observer can weigh at the bedside [46].

Model performance is now consistently reported in the AUC 0.80–0.88 range. A deep-learning model using a small number of smartphone photographs of the face and neck in four standard views achieved AUROC 0.81–0.88 with F1-scores of 0.72–0.81, and gradient-weighted class activation mapping localised the discriminatory information to the neck and chin—anatomically coherent with the ultrasound findings above [47]. Multi-view metric learning architectures report an accuracy of 77.9% with a sensitivity of 82.5% [48]. The approach has since been extended from two-dimensional photographs to three-dimensional facial geometry: in a prospective study of 398 patients, pre-operative face scans fitted to an established non-clinical facial model predicted difficult facemask ventilation at least as well as the DIFFMASK score (optimism-corrected area under the curve 0.74 versus 0.73), and enriching that score with three interpretable shape features raised it to 0.76, with colour-mapped meshes localising the predictive information to the nose, lower mandible, neck region and facial convexity [49]. The authors describe this as digital phenotyping, and the term is apt: what such a model recovers is not a threshold but an individual morphology—the same object that the phenotype-specific pathways of Section 5 approach clinically. Models built on routinely collected clinical variables in defined surgical populations reach areas under the curve of up to 0.848 in the test set [50]. Voice analysis and multiparametric assessment are emerging as further input channels [8,51], and analogous machine-learning screening models are advancing rapidly in OSA [52] and paediatric anaesthesia [53].

Two caveats must be stated plainly, and the most recent narrative synthesis of AI in airway management states them well: positive predictive value remains the fundamental limitation—improved relative to bedside tests, but still imperfect—and the risks of clinician deskilling, opacity of “black box” models and insufficient external validation are real [8]. To these should be added a methodological concern that recurs across the primary literature: several models are trained after artificially balancing difficult and non-difficult cases to a 1:1 ratio [47]. This is defensible for model development but renders the reported predictive values inapplicable to a clinical population in which the event occurs in fewer than one patient in ten, unless calibration is subsequently restored. Reported discrimination and reported clinical usefulness are not the same quantity.

### 4.4. The Operator and the Environment as Data

A precision framework that models only the patient will systematically mispredict. The Cochrane review of videolaryngoscopy in adults found reduced failed intubation (RR 0.43, 95% CI 0.30–0.61), reduced hypoxaemia (RR 0.25, 95% CI 0.12–0.50), improved first-attempt success (RR 1.10, 95% CI 1.05–1.15) and markedly improved glottic view (RR 0.14 for Cormack–Lehane 3/4 views) compared with direct laryngoscopy [54]. A patient classified as “difficult” for direct laryngoscopy may be unremarkable with a hyperangulated blade and an experienced operator, and formidable for a trainee working night-time hours in an emergency department [55]. Operator experience, device availability, time of day, assistance and setting are therefore not confounders to be adjusted away but legitimate inputs to an individualised risk estimate—and, unlike most patient factors, they are modifiable—. The magnitude of the operator term is measurable: in a high-fidelity simulation study of AI-assisted videolaryngoscopy in 99 practitioners, basic procedural competency was reached at the fourth attempt, glottic visualisation continued to improve through the tenth, and anaesthesia residents outperformed participants without prior intubation experience in success rate (98% versus 93%), glottic view and time to intubation [56]. A risk model that treats “the operator” as a constant is discarding a variable of comparable weight to several of the patient factors it retains. This is now formalised in guidance: ESAIC recommends that the minimum set of preoperative airway assessment include, alongside anatomical tests, the evaluation of physiology, environment, devices, and individual and team expertise, and reframes the objective as airway management planning rather than difficult airway prediction [6].

## 5. Phenotype-Specific Pathways

The claim of this section is that “difficult airway” is not a single condition. The populations below differ not merely in the prevalence of difficulty but in its mechanism, in which test performs, and in which intervention mitigates it. Assessment should differ accordingly—a principle for which guideline support already exists, ESAIC recommending that the minimum set of airway assessment tests vary between patients according to the specific underlying pathology [6]. What follows is an attempt to specify what that variation should look like.

### 5.1. Obesity and Obstructive Sleep Apnoea

Patients living with obesity are consistently overrepresented in airway complication series, and the Society for Obesity and Bariatric Anaesthesia has issued 43 dedicated best-practice recommendations [57]. The defining feature of this phenotype is dissociation between the domains of difficulty: laryngoscopy is often unremarkable, whereas mask ventilation is difficult in 14.4% (versus 6.1% overall) [18], and the safe apnoea time is drastically shortened. OSA independently confers a three- to four-fold higher risk of difficult intubation, difficult mask ventilation or both [58], and unrecognised OSA risk is common in surgical populations: in one prospective series 54.2% of patients screened positive on STOP-BANG, and—tellingly for the argument of this section—a positive screen was significantly associated with predictors of difficult mask ventilation but not with predictors of difficult intubation [59]. The wider anaesthetic literature on obesity converges on the same conclusion—that the perioperative risk of this phenotype is respiratory and positional rather than laryngoscopic [60]—and descriptions of the “bariatric airway” have long emphasised planning, positioning and oxygenation over device selection [61].

Practical consequences: neck circumference adds predictive information not captured by body mass index and can be incorporated into composite scoring in this group [24]; among bedside tests, the ULBT and thyromental height retain—and in some series exceed—their usual performance, with the ULBT achieving positive likelihood ratios of 6.35–9.47 versus 3.16–3.21 for the MMT in morbidly obese patients [62] and a sensitivity of 93.3% in a logistic-regression analysis identifying thyromental height, body mass index, hyomental distance ratio and the ULBT as independent predictors [63]; ultrasound retains discriminatory value [40]; positioning (ramped, head-elevated laryngoscopy position) and preoxygenation strategy matter more than device choice; and cricothyroid membrane identification by ultrasound should be considered pre-induction in patients with a high neck circumference [42]. Assessment strategies, device selection and adjuncts specific to the OSA population have been appraised in detail and should inform local protocols [64].

### 5.2. The Obstetric Patient

The obstetric airway combines anatomical change (mucosal oedema, breast tissue, weight gain), physiological vulnerability (reduced functional residual capacity, increased oxygen consumption, rapid desaturation), aspiration risk and, frequently, time pressure [65,66]. Critically, airway status is not static: it evolves across pregnancy and labour, so a single antenatal assessment has a limited shelf-life.

The literature here is unusually explicit about individualisation. Antenatal planning is recommended for patients with identified risk factors, encompassing assessment of clinical characteristics, the equipment and personnel available out-of-hours, and—where these cannot be assured—the option of elective caesarean delivery to avoid emergency airway management; awake tracheal intubation is advised where safe airway management after induction cannot be assured [65]. Ultrasound airway parameters have been specifically evaluated in pregnancy, where sonographic measurements differ significantly from those of non-pregnant women and where pregnancy itself, hyoid bone visibility and the pre-epiglottic space to epiglottis–vocal cord ratio emerged as independent predictors of a difficult airway [39]; the cricothyroid membrane data discussed above derive from labouring women [42]. Contemporary syntheses of the obstetric airway increasingly frame the problem in terms of human factors—teamwork, training and system design under time pressure—rather than of anatomy alone [67], and national adaptations of international algorithms exist for obstetric practice [68]. Broader clinical updates on caesarean delivery situate airway planning within the wider obstetric anaesthetic pathway [69], while narrative reviews of obstetric emergencies address the failed obstetric airway alongside the other time-critical scenarios with which it competes for the same team [66].

### 5.3. Head and Neck Oncology and Post-Radiotherapy Anatomy

This is the highest-risk phenotype and the least well served by conventional bedside testing, because the relevant pathology lies deeper than the surface being inspected. In a series of 200 consecutive head and neck cancer operations, airway management required awake fibreoptic intubation in 15% and tracheostomy under local anaesthesia in 9%, with three emergency tracheostomies, all in locally advanced laryngeal cancer [70]. Previous neck radiotherapy is the single strongest reported risk factor for difficult mask ventilation (OR 5.0) [18]; in a cohort of irradiated head and neck patients, a difficult airway was encountered in 50%, alongside a 39% prevalence of clinical or subclinical hypothyroidism, illustrating how the relevant preoperative work-up in this phenotype extends beyond the airway itself [71].

For this phenotype the assessment hierarchy inverts: history (tumour site, stage, prior radiotherapy, prior surgery, stridor, positional dyspnoea) and existing cross-sectional imaging dominate, endoscopic evaluation is often decisive, and generic bedside tests are of secondary value—although in patients undergoing head and neck surgery the MMT, the Wilson risk score, the ULBT and mouth opening do remain associated with both difficult laryngoscopy and difficult intubation [72]. Composite indices have been applied prospectively as decision rules: in a tertiary cancer centre, an El-Ganzouri risk index above 7 triggered awake intubation, with fibreoptic bronchoscopy used in 54% and videolaryngoscopy in 42.6% of cases [73]. Thyroid surgery—where difficulty affects 7.21% (95% CI 6.57–7.91%), with Mallampati ≥ 3 (OR 4.75), short thyromental distance (OR 3.64), reduced interincisor gap (OR 2.57) and tracheal deviation (OR 2.06) as predictors [74]—shows how a disease-specific parameter (tracheal deviation) can enter the model alongside generic ones; contemporary perioperative frameworks for thyroid surgery accordingly combine nasendoscopy, airway ultrasound and the neck circumference-to-thyromental distance ratio with first-line videolaryngoscopy, rather than relying on generic bedside scoring [75]. Free-flap reconstructive surgery adds prolonged duration, goal-directed haemodynamic management and postoperative airway compromise to the planning problem [76], and the shared airway imposes its own oxygenation constraints: high-flow nasal oxygen (HFNO), jet ventilation and ultra-narrow tracheal tube ventilation each trade surgical access against carbon dioxide clearance and fire risk, so that the oxygenation strategy—like the intubation strategy—must be selected case by case rather than by protocol [77].

### 5.4. The Critically Ill and Emergency Patient: Physiological Difficulty

In critical illness, the binding constraint is frequently not anatomy but physiological reserve. The concept of the physiologically difficult airway—hypoxaemia, haemodynamic instability, metabolic acidosis, right ventricular failure—is now formalised in consensus recommendations from the Society for Airway Management [78] and in an international Delphi process that reached stable consensus on 53 of 61 statements, explicitly including pregnancy and obesity as physiologically difficult airway states [79,80].

The magnitude of the problem is quantified by the INTUBE study: peri-intubation cardiovascular instability or collapse occurred in 1199 of 2760 critically ill patients (43.4%) and was associated with an adjusted odds ratio for ICU mortality of 2.47 (95% CI 1.72–3.55) [81]. Two distinct questions therefore have to be answered in this population, and they should not be conflated. The first is whether tracheal intubation will be technically difficult; here the MACOCHA score remains the best-validated instrument, having been developed and externally validated specifically to predict difficult intubation in critically ill patients [25]. Its inputs include physiological variables—severe hypoxaemia and coma—but the outcome it predicts is procedural difficulty, not haemodynamic instability. The second is whether the patient will tolerate the attempt, for which no comparably validated instrument exists; risk is currently estimated from the pre-induction physiological state itself—systolic pressure, heart rate and oxygen saturation—and from the choice of induction agent, all of which were independently associated with peri-intubation collapse in INTUBE [81]. A low MACOCHA score therefore does not license a rapid, unsupported induction in a shocked patient: anatomical reassurance and physiological reassurance are separate judgements, and only the first is currently scored.

Correspondingly, the mitigating interventions are physiological. The PREOXI trial demonstrated that preoxygenation with non-invasive ventilation (NIV) reduced hypoxaemia during emergency intubation from 18.5% to 9.1% (difference −9.4 percentage points, 95% CI −13.2 to −5.6), with cardiac arrest in 0.2% versus 1.1% [82]; a network meta-analysis of preoxygenation strategies contextualises the relative positions of facemask, high-flow nasal oxygen and non-invasive ventilation [83], and the physiological rationale for non-invasive positive pressure ventilation—alveolar recruitment, reduced shunt, preserved functional residual capacity—together with the patient categories in which it is contraindicated, has been reviewed in detail [84]. Pharmacological choices follow the same phenotype logic: in contemporary rapid sequence induction the dominant risks in the critically ill are desaturation and haemodynamic collapse rather than aspiration, favouring rocuronium over succinylcholine and ketamine or etomidate over propofol, with first-pass success—currently around 84%—as the outcome that governs complication rates [85]. Contemporary syntheses of evidence-based emergency intubation converge on bundled, checklist-driven, team-based approaches [86,87,88].

### 5.5. Paediatric and Syndromic Patients

Children are not small adults, and the paediatric evidence makes the case for phenotype-specific pathways particularly clearly. In the Pediatric Difficult Intubation registry, first-attempt success in children with difficult airways was 3% with direct laryngoscopy, 54% with fibreoptic bronchoscopy and 55% with indirect videolaryngoscopy; complications occurred in 20% of encounters (3% severe, cardiac arrest in 2%) and were associated with more than two intubation attempts, weight below 10 kg and short thyromental distance [89]. A subsequent registry analysis found similar first-attempt success for sedation versus general anaesthesia (48.3% vs. 47.9%), although 27.6% of sedation cases required conversion [90]. Joint ESAIC–BJA guidelines now cover neonates and infants specifically [91], with narrative syntheses emphasising human factors alongside technique [92], and a randomised trial has demonstrated superiority of videolaryngoscopy for urgent intubation of newborn infants [93].

The precision message is direct: in children, the decisive variables are age, weight, syndromic diagnosis and number of attempts—not adult bedside scores, which are frequently unobtainable in an uncooperative child.

### 5.6. Overlapping Phenotypes

Real patients rarely occupy a single category: a woman with obesity and obstructive sleep apnoea may present for emergency caesarean delivery, a patient with a post-radiotherapy neck may require intubation in septic shock. The phenotypes above are therefore not compartments but markers of where risk is concentrated, and three rules make them usable when several apply at once.

1. Assessments are additive, not alternative. The obese patient with a previously irradiated neck requires both the ultrasound CTM marking indicated by the first phenotype and the endoscopic and cross-sectional imaging review indicated by the second. Adding an assessment costs minutes; omitting one costs the information it would have given.
2. The most constraining mitigation governs. Where two phenotypes suggest different strategies, the one that tolerates least deterioration should prevail. In the obese obstetric patient, this means the obstetric rule—awake tracheal intubation if safe post-induction management cannot be assured—rather than the more permissive default for obesity alone.
3. The three risk domains should be assessed and documented separately even when the phenotype label is single. Anatomical difficulty, physiological vulnerability and contextual constraint are independent, are predicted by different variables and are mitigated by different interventions; a patient may be reassuring in one domain and dangerous in another; Section 5.4 illustrates how easily a favourable anatomical score can be mistaken for overall safety. Recording an explicit judgement in each domain—rather than a single global impression of “difficult” or “not difficult”—is the smallest change to current practice that this review proposes, and the one most likely to survive the overlap between categories. No validated composite score exists for this purpose, and none is implied here; what is proposed is structured documentation, not quantification. Table 3 summarises the resulting pathways.

## 6. Translating Stratification into Strategy: Alignment with the 2025 Difficult Airway Society Guidelines

Risk stratification is worth performing only if it changes management. Two 2025 documents provide the natural downstream structure for an individualised estimate: the ESAIC guidelines, which govern what should be assessed preoperatively and require that the assessment be tailored to the patient [6], and the DAS guidelines, which govern what should be done when difficulty nevertheless declares itself [5]. The translation operates at five decision points.

Awake versus post-induction airway management. This remains the single decision most sensitive to accurate risk estimation. Established clinical warning signs—stridor, post-radiotherapy fibrosis, a class 3 upper lip bite test, a history of previous difficulty—should weigh heavily in favour of an awake technique [13,70,71]. Sonographic findings such as a markedly increased distance from skin to epiglottis are best regarded as supportive rather than decisive: their discriminatory performance is established, but, as discussed in Section 4.1, evidence that acting on them alters management or outcome is not, and ESAIC has accordingly declined to recommend instrumental airway assessment as routine [6]. Awake tracheal intubation nonetheless remains underused relative to guideline recommendations, partly because the sedation regimen is perceived as difficult: over thirty regimens have been described with no clear superiority, and adequate topicalisation with lidocaine matters more than the choice of hypnotic—any regimen being better than none [94]. Stratification is of little value if the technique it selects is one the team is reluctant to perform (Section 7.4).

First-line device. Given the demonstrated superiority of videolaryngoscopy across adult populations [54], the question in a precision framework is not whether to have a videolaryngoscope available but for whom it should be the planned first device, and which blade geometry the predicted anatomy favours. The setting and the operator modify this calculus: in a review of six studies and 4525 patients intubated during in-hospital cardiac arrest, first-pass success improved with videolaryngoscopy only when the intubator was a less experienced clinician [95]—a direct demonstration that the same device carries a different expected benefit depending on who is holding it. This reframing—from the selection of a device to a patient-centred, oxygenation-focused strategy in which assessment, prediction and the timing of transition between techniques matter more than the instrument itself—is the organising principle of recent practical syntheses of safe airway management [96].

Oxygenation strategy. Preoxygenation should be prescribed to phenotype rather than applied uniformly: non-invasive ventilation for the hypoxaemic critically ill patient [82,83], ramped positioning and extended preoxygenation in obesity [57], recognition of rapid desaturation in pregnancy [65].

Team and setting. The Delphi consensus on the physiologically difficult airway recommends an intubation team of at least three providers including two airway operators, a designed checklist and haemodynamic optimisation before intubation [79]. Assembling this for every patient is not feasible; assembling it for stratified high-risk patients is.

Front-of-neck access readiness. Ultrasound marking of the cricothyroid membrane should be targeted at patients in whom palpation is predictably unreliable: high neck circumference, obesity, post-radiotherapy necks, labouring women [42].

A workable operational rule is that assessment intensity should be proportional to pre-test probability: a brief structured bedside assessment for unselected low-risk patients; the addition of targeted ultrasound and composite scoring at intermediate risk; and full multimodal evaluation including existing imaging, endoscopic assessment and multidisciplinary planning at high risk. This is the same tiering logic already used for cardiac risk assessment before non-cardiac surgery, applied to an organ system in which it has not been formalised.

## 7. Barriers, Equity and Validation Requirements

### 7.1. Validation and Calibration

Most published prediction models for the difficult airway report discrimination in a development cohort and stop there. Deployment requires three further properties: external validation in a population with the intended case mix; calibration, so that a predicted probability of 20% corresponds to an observed frequency of 20%; and demonstrated clinical utility, ideally by decision-curve analysis or a prospective impact study. The DIFFICAIR trial showed that an instrument with acceptable statistical properties may still fail to change outcomes when implemented [26].

### 7.2. Algorithmic Fairness

Models that ingest facial images, voice or anthropometry are exposed to a specific and well-documented failure mode. Scoping reviews of racial bias in clinical machine learning [97] and analyses of bias mechanisms in imaging AI—including “shortcut” learning, in which a model exploits a spurious correlate rather than the intended signal [98]—establish that such bias is common rather than exceptional. Race- and ethnicity-related bias has been demonstrated in risk prediction models in other specialties [99], mitigation methods are an active field [100,101], and fairness has been shown to drift over time after deployment, requiring ongoing surveillance rather than one-off certification [102].

For airway models specifically, the exposures are concrete: skin tone and lighting in facial image datasets; sex differences in neck anatomy and soft-tissue distribution; ethnic variation in craniofacial morphology; and the exclusion of edentulous, non-cooperative or non-English-speaking patients from training cohorts. Performance stratified by these characteristics should become a minimum reporting standard.

### 7.3. Implementation, Regulation and Training

Three further barriers deserve mention. Workflow integration: an assessment that adds five minutes per patient will not survive a high-throughput list unless it is targeted at those who benefit. Regulation: models intended to inform clinical decisions fall within the scope of medical device and AI regulatory frameworks, with corresponding requirements for transparency, post-market surveillance and human oversight [8]. Training and access: ultrasound-based assessment presupposes trained operators and available probes, and an intervention that improves outcomes only in well-resourced institutions widens rather than narrows the equity gap.

### 7.4. Skills, and the Deliverability of the Plan

A framework for deciding what to do is worth nothing if the technique it selects cannot be performed. This is the most important practical limitation of everything proposed above, and it is not a limitation of prediction but of preparedness.

Two competences carry the plan when stratification identifies high risk. The first is flexible bronchoscopic intubation, which remains the technique of choice where anatomy is distorted by tumour, previous surgery or radiotherapy and where a videolaryngoscope may not be usable at all [103]. The second is emergency front-of-neck access, the final common pathway when oxygenation fails. Both are precisely the skills most exposed to decay, and for a paradoxical reason: as videolaryngoscopy has made routine intubation easier and more reliable [54], the clinical opportunities to practise the alternatives have become correspondingly rare. The same concern has been raised about de-skilling in direct laryngoscopy as videolaryngoscopy becomes the default [104]. Awake tracheal intubation illustrates the consequence: it is recommended by guidelines for the anticipated difficult airway yet remains underused, in part because teams are not confident in performing it [94], and the registry data are consistent with this—in children with difficult airways, first-attempt success with flexible bronchoscopy was 54%, no better than with videolaryngoscopy, in a setting where operators were experienced enough to be enrolled at all [89].

The implication for the framework proposed here is direct. Contextual risk, which Section 5.6 asks clinicians to document alongside anatomical and physiological risk, includes the competence actually available at the time and place of the procedure—not the competence available in principle to the institution. Where a stratified assessment indicates awake intubation or standby front-of-neck access and the skills to deliver them are not immediately present, the correct response is to change the time, the team or the place, not the assessment. Structured training addresses the same gap upstream: cadaveric and simulation-based courses improve confidence and translate into clinical practice for awake intubation [105], and artificial intelligence, whatever its eventual role in prediction, is likely to prove more immediately useful in accelerating procedural training, as the learning-curve data in Section 4.4 suggest [56]. Prediction and preparedness are complementary; neither substitutes for the other.

## 8. Future Directions

Four priorities follow from this analysis. First, outcome standardisation: prediction studies should adopt a common core outcome set—ideally centred on first-attempt success and on patient-centred harm rather than on Cormack–Lehane grade, which is an intermediate measure of glottic view rather than of difficulty. The MASCAN classification shows what this looks like for a single domain [22]; the equivalent work for difficult laryngoscopy and difficult intubation remains to be done. Second, prospective multicentre registries capturing patient, operator, device and environmental variables in parallel, since the latter are systematically absent from current datasets. Third, integration of anatomical and physiological difficulty into a single risk estimate, as clinicians already do implicitly and no published model yet does explicitly. Fourth, impact studies rather than accuracy studies: the field has ample evidence that measurements correlate with difficulty and almost none that acting on them improves outcomes.

## 9. Clinical Implications

The argument of this review can be reduced to seven practice points. The first three require no new equipment and no new evidence; the remainder are proposals that follow from the evidence reviewed but have not themselves been tested prospectively.

1. A normal bedside examination is not reassurance. The modified Mallampati test detects fewer than four in ten difficult intubations, and in routine practice 93% of difficult intubations occur in patients in whom difficulty was not anticipated [3,12]. A normal bedside examination does not exclude difficulty and should not reduce baseline readiness for difficult-airway rescue; positive findings, or phenotype-specific risk factors identified elsewhere in the assessment, should prompt escalation of the plan.
2. Record three judgements, not one. Anatomical difficulty, physiological vulnerability and contextual constraint—operator, device, assistance, time of day—are predicted by different variables and mitigated by different actions. Documenting them separately, instead of a single global impression of “difficult” or “not difficult”, is the smallest change to current practice proposed here.
3. Ask what the test is for. A test validated for laryngoscopy does not predict difficult facemask ventilation, and the risk factors for the latter—previous neck radiotherapy, increased neck circumference, obstructive sleep apnoea, beard, obesity—are largely different [18]. Where mask ventilation is the vulnerable step, as in obesity, that is the domain to assess.
4. Match the intensity of assessment to the pre-test probability. For unselected low-risk patients, a brief structured bedside assessment. At intermediate risk, the addition of composite scoring and, where available, targeted ultrasound. At high risk, review of existing cross-sectional imaging, endoscopic assessment where indicated, and multidisciplinary planning before the day of surgery.
5. Mark the cricothyroid membrane in patients whose necks cannot be palpated reliably. Digital palpation identified the membrane in only 39% of obese parturients [42]. In precisely the patients most likely to need front-of-neck rescue, the landmark is least likely to be found without ultrasound, and marking it costs a minute before induction.
6. Check that the plan can be delivered here, tonight, by this team. A stratified assessment that selects awake intubation or standby front-of-neck access is only as good as the competence immediately available. Where the skills are not present, change the time, the team or the place rather than the assessment [102].
7. In critical illness, do not read anatomical reassurance as physiological safety. MACOCHA predicts difficult intubation, not cardiovascular collapse [25]. Haemodynamic and oxygenation reserve must be appraised separately and optimised before induction, and the team assembled accordingly [79,80].

## 10. Conclusions

For four decades, preoperative airway assessment has been run as a universal screening programme, and it performs as such programmes do when the event is uncommon: it is specific but insensitive, most of its alarms are false, and most of the events it exists to detect arrive unannounced. Refining individual bedside tests further is unlikely to change this, because the limit lies in the design of the task rather than in the tests.

What is needed is not a better test but a different question. Instead of asking whether a patient is difficult, the clinician should ask how much anatomical, physiological and contextual risk this particular patient carries, and how much assessment that risk justifies. Point-of-care ultrasound already offers more information than inspection alone, although whether acting on it improves outcomes has still to be shown. Machine learning is promising but needs external validation, calibration and demonstrated fairness before deployment. The phenotype-oriented framework proposed here requires no new technology and could be evaluated prospectively now.

The 2025 ESAIC and Difficult Airway Society guidelines have improved what happens when prediction fails. The task ahead is to make it fail less often.

## Figures and Tables

**Table 3:** Proposed phenotype-oriented framework for airway assessment. The table is a synthesis of the evidence discussed in Section 5 and is offered as a hypothesis-generating framework, not as a validated instrument: the individual components are supported to differing degrees, but these pathways have not been prospectively evaluated as integrated assessment strategies, and the same evidential gap identified in Section 8—that measurements correlate with difficulty while acting on them has rarely been shown to improve outcome—applies to the framework as a whole. Risk domains are anatomical, physiological and contextual (operator, device, setting, urgency). The phenotypes are not mutually exclusive; where more than one applies, the assessments are additive and the most constraining mitigation governs (see Section 5.6).

Phenotype	Predominant Risk Domain(s)	Proposed Assessment Emphasis	Proposed Mitigation
Obesity/OSA	Anatomical (mask seal, tissue redundancy) and physiological (reduced safe apnoea time)	Neck circumference; OSA screening; ultrasound; CTM marking	Ramped position; NIV/HFNO preoxygenation; early videolaryngoscopy [18,40,42,57,58]
Obstetric	Physiological (reduced reserve, aspiration risk) and contextual (urgency, out-of-hours); anatomy evolves across pregnancy and labour	Serial (not single) assessment; antenatal multidisciplinary planning	Elective delivery planning; awake intubation if safety not assured [39,65,66]
Head and neck cancer/post-radiotherapy	Anatomical, fixed and deep to the inspected surface	History, existing CT/MRI, endoscopic evaluation	Awake fibreoptic intubation; elective tracheostomy; surgical standby [18,70,71,74]
Critically ill/emergency	Physiological vulnerability; anatomical difficulty also common and separately predicted	MACOCHA for anatomical and procedural difficulty; separate appraisal of haemodynamic and oxygenation reserve	NIV preoxygenation; haemodynamic optimisation; two operators; checklist [25,78,79,81,82]
Paediatric/syndromic	Anatomical (age-, weight- and syndrome-specific) and contextual (operator experience)	Syndromic diagnosis; weight; prior airway history	Early indirect technique; attempt limitation; videolaryngoscopy [89,90,91,93]
Unselected adult, low risk	Low pre-test probability in all three domains	Brief bedside assessment	Standard care; videolaryngoscopy availability [11,13,54]

## Data Availability

No new data were created or analysed in this study. Data sharing is not applicable to this article.

## Supplementary Materials

The following supporting information can be downloaded at: https://www.mdpi.com/article/10.3390/jpm16100532/s1, Supplementary Material S1: Search strategy.

## Abbreviations

AI, artificial intelligence; AUC, area under the curve; AUROC, area under the receiver operating characteristic curve; CTM, cricothyroid membrane; DAS, Difficult Airway Society; DSE, distance from skin to epiglottis; HFNO, high-flow nasal oxygen; ICU, intensive care unit; MMT, modified Mallampati test; NAP4, Fourth National Audit Project; NIV, non-invasive ventilation; OSA, obstructive sleep apnoea; POCUS, point-of-care ultrasound; ULBT, upper lip bite test.

## References

[1] Cook T.M., Woodall N., Frerk C. (2011). Major complications of airway management in the UK: Results of the Fourth National Audit Project of the Royal College of Anaesthetists and the Difficult Airway Society. Part 1: Anaesthesia. Br. J. Anaesth..

[2] Cook T.M., Woodall N., Harper J., Benger J. (2011). Major complications of airway management in the UK: Results of the Fourth National Audit Project of the Royal College of Anaesthetists and the Difficult Airway Society. Part 2: Intensive care and emergency departments. Br. J. Anaesth..

[3] Norskov A.K., Rosenstock C.V., Wetterslev J., Astrup G., Afshari A., Lundstrom L.H. (2015). Diagnostic accuracy of anaesthesiologists’ prediction of difficult airway management in daily clinical practice: A cohort study of 188 064 patients registered in the Danish Anaesthesia Database. Anaesthesia.

[4] Weller J.M. (2024). Failed intubation: Anaesthesia’s Achilles’ heel. Br. J. Anaesth..

[5] Ahmad I., El-Boghdadly K., Iliff H., Dua G., Higgs A., Huntington M., Mir F., Nouraei S.A.R., O’Sullivan E.P., Patel A. (2025). Difficult Airway Society 2025 guidelines for management of unanticipated difficult tracheal intubation in adults. Br. J. Anaesth..

[6] Lamperti M., Romero C.S., Guarracino F., Cammarota G., Vetrugno L., Tufegdzic B., Lozsan F., Macias Frias J.J., Duma A., Bock M. (2025). Preoperative assessment of adults undergoing elective noncardiac surgery: Updated guidelines from the European Society of Anaesthesiology and Intensive Care. Eur. J. Anaesthesiol..

[7] Chen H., Zheng Y., Fu Q., Li P. (2024). A review of the current status and progress in difficult airway assessment research. Eur. J. Med. Res..

[8] Sorbello M., La Via L., Paternò D.S., Tutino S., Lo Giudice E.C., Lentini M., Maniaci A., Pappalardo F. (2026). Artificial intelligence in airway management: A narrative review. Br. J. Anaesth..

[9] Paternò D.S., La Via L., Lo Giudice E., Lentini M., Maniaci A., Bonaccorso A.M., Moltisanti R., Putaggio A., Pappalardo F., Sorbello M. (2026). Point-of-Care Ultrasound in Airway Management. J. Clin. Med..

[10] Baethge C., Goldbeck-Wood S., Mertens S. (2019). SANRA-a scale for the quality assessment of narrative review articles. Res. Integr. Peer Rev..

[11] Roth D., Pace N.L., Lee A., Hovhannisyan K., Warenits A.M., Arrich J., Herkner H. (2018). Airway physical examination tests for detection of difficult airway management in apparently normal adult patients. Cochrane Database Syst. Rev..

[12] Wang Z., Jin Y., Zheng Y., Chen H., Feng J., Sun J. (2024). Evaluation of preoperative difficult airway prediction methods for adult patients without obvious airway abnormalities: A systematic review and meta-analysis. BMC Anesthesiol..

[13] Detsky M.E., Jivraj N., Adhikari N.K., Friedrich J.O., Pinto R., Simel D.L., Wijeysundera D.N., Scales D.C. (2019). Will This Patient Be Difficult to Intubate?: The Rational Clinical Examination Systematic Review. JAMA.

[14] Faramarzi E., Soleimanpour H., Khan Z.H., Mahmoodpoor A., Sanaie S. (2018). Upper lip bite test for prediction of difficult airway: A systematic review. Pak. J. Med. Sci..

[15] Carvalho C.Í.C.Í., Santos Neto J.M., Orange F.V.A. (2021). Predictive performance of thyromental height for difficult laryngoscopies in adults: A systematic review and meta-analysis. Braz. J. Anesthesiol..

[16] Chen W., Tian T., Li X., Jiang T., Xue F. (2022). Use of the Thyromental Height Test for Prediction of Difficult Laryngoscopy: A Systematic Review and Meta-Analysis. J. Clin. Med..

[17] Honarmand A., Safavi M., Yaraghi A., Attari M., Khazaei M., Zamani M. (2015). Comparison of five methods in predicting difficult laryngoscopy: Neck circumference, neck circumference to thyromental distance ratio, the ratio of height to thyromental distance, upper lip bite test and Mallampati test. Adv. Biomed. Res..

[18] Hung K.C., Chuang M.H., Kang F.C., Chang Y.J., Lin C.M., Yu C.H., Chen I.W., Sun C.K. (2023). Prevalence and risk factors of difficult mask ventilation: A systematic review and meta-analysis. J. Clin. Anesth..

[19] Adamus M., Jor O., Vavreckova T., Hrabalek L., Zapletalova J., Gabrhelik T., Tomaskova H., Janout V. (2011). Inter-observer reproducibility of 15 tests used for predicting difficult intubation. Biomed. Pap..

[20] Rosenstock C., Gillesberg I., Gätke M.R., Levin D., Kristensen M.S., Rasmussen L.S. (2005). Inter-observer agreement of tests used for prediction of difficult laryngoscopy/tracheal intubation. Acta Anaesthesiol. Scand..

[21] Wang L.Y., Zhang K.D., Zhang Z.H., Zhang D.X., Wang H.L., Qi F. (2022). Evaluation of the reliability of the upper lip bite test and the modified mallampati test in predicting difficult intubation under direct laryngoscopy in apparently normal patients: A prospective observational clinical study. BMC Anesthesiol..

[22] Wünsch V.A., Bommes H., Piltan M., Germer S., Gundler C., Dohrmann T., Köhl V., Dankert A., Krause L., Zöllner C. (2026). Prospective development and validation of an objective classification for difficult facemask ventilation: The MASCAN score. Anaesthesia.

[23] Bicalho G.P., Bessa R.C., Cruvinel M.G.C., Carneiro F.S., Castilho J.B., Castro C.H.V. (2021). A prospective validation and comparison of three multivariate models for prediction of difficult intubation in adult patients. Braz. J. Anesthesiol..

[24] Corrente A., Fiore M., Colandrea S., Aurilio C., Passavanti M., Pota V., Sansone P., Diana G., Signoriello G., Pace M. (2020). A new simple score for prediction of difficult laryngoscopy: The EL.GA+ score. Anaesthesiol. Intensive Ther..

[25] De Jong A., Molinari N., Terzi N., Mongardon N., Arnal J.M., Guitton C., Allaouchiche B., Paugam-Burtz C., Constantin J.M., Lefrant J.Y. (2013). Early identification of patients at risk for difficult intubation in the intensive care unit: Development and validation of the MACOCHA score in a multicenter cohort study. Am. J. Respir. Crit. Care Med..

[26] Norskov A.K. (2016). Preoperative airway assessment—Experience gained from a multicentre cluster randomised trial and the Danish Anaesthesia Database. Dan. Med. J..

[27] Carsetti A., Sorbello M., Adrario E., Donati A., Falcetta S. (2022). Airway Ultrasound as Predictor of Difficult Direct Laryngoscopy: A Systematic Review and Meta-analysis. Anesth. Analg..

[28] Soni V.M., Pappu A., Zarabi S., Khalil C., You-Ten K.E., Siddiqui N., Wong D.T., Chan V., Li Q., Huzsti E. (2025). Point-of-care ultrasound of the upper airway in difficult airway management: A systematic review and meta-analysis. Anaesthesia.

[29] Giordano G., Alessandri F., Zulian A., Bilotta F., Pugliese F. (2023). Pre-operative ultrasound prediction of difficult airway management in adult patients: A systematic review of clinical evidence. Eur. J. Anaesthesiol..

[30] Benavides-Zora D., Jaramillo M.C., Townsley M.M., Franco V., González S., Hoyos C., Cerón J., Arias-Botero J.H., Sondekoppam R.V., Kalagara H. (2023). Diagnostic Performance of Airway Ultrasound for the Assessment of Difficult Laryngoscopy: A Systematic Review and Meta-Analysis. J. Cardiothorac. Vasc. Anesth..

[31] Sotoodehnia M., Rafiemanesh H., Mirfazaelian H., Safaie A., Baratloo A. (2021). Ultrasonography indicators for predicting difficult intubation: A systematic review and meta-analysis. BMC Emerg. Med..

[32] Gomes S.H., Simões A.M., Nunes A.M., Pereira M.V., Teoh W.H., Costa P.S., Kristensen M.S., Teixeira P.M., Pêgo J.M. (2021). Useful Ultrasonographic Parameters to Predict Difficult Laryngoscopy and Difficult Tracheal Intubation-A Systematic Review and Meta-Analysis. Front. Med..

[33] Yuan J., Ye H., Tan X., Zhang H., Sun J. (2024). Determinants of difficult laryngoscopy based on upper airway indicators: A prospective observational study. BMC Anesthesiol..

[34] Pillai A., Arora P., Kabi A., Chauhan U., Asokan R., Akhil P., Shankar T., Lalneiruol D.J., Baid H., Chawang H. (2024). The diagnostic accuracy of point-of-care ultrasound parameters for airway assessment in patients undergoing intubation in emergency department-an observational study. Int. J. Emerg. Med..

[35] Parameshwar S.K., Karna S.T., Waindeskar V., Kumar H., Singh P., Saigal S. (2023). Accuracy of Sonographic Airway Parameters in Difficult Laryngoscopy Prediction: A Prospective Observational Cohort Study from Central India. Turk. J. Anaesthesiol. Reanim..

[36] Adi O., Fong C.P., Sum K.M., Ahmad A.H. (2020). Usage of airway ultrasound as an assessment and prediction tool of a difficult airway management. Am. J. Emerg. Med..

[37] Petrișor C., Trancă S., Szabo R., Simon R., Prie A., Bodolea C. (2020). ClinicalUltrasound Measurements of Hyomental Distance Ratio for the Prediction of Difficult Airway in Patients with and without Morbid Obesity. Diagnostics.

[38] Petrisor C., Szabo R., Constantinescu C., Prie A., Hagau N. (2018). Ultrasound-based assessment of hyomental distances in neutral, ramped, and maximum hyperextended positions, and derived ratios, for the prediction of difficult airway in the obese population: A pilot diagnostic accuracy study. Anaesthesiol. Intensive Ther..

[39] Vajanthri S.Y., Mohammed S., Kumar M., Chhabra S., Bhatia P., Kamal M., Paliwal B. (2022). Evaluation of ultrasound airway assessment parameters in pregnant patients and their comparison with that of non-pregnant women: A prospective cohort study. Int. J. Obstet. Anesth..

[40] El-Tawansy A., Mohamed Salama Elnajar A., Abdel Baky Mahmoud H., Ibrahim Amin M., Abd Elmohsen Bedewy A. (2024). Validity of Airway Ultrasound in Correlation with Cormack-Lehane Grading in Obese Patients: A Cross-Sectional Study. Anesth. Pain Med..

[41] Kahlon S., Jain D., Bhardwaj N., Gandhi K., Jafra A. (2022). Ultrasound Evaluation to Predict Difficult Laryngoscopy in Children below 2 Years. Indian J. Pediatr..

[42] You-Ten K.E., Desai D., Postonogova T., Siddiqui N. (2015). Accuracy of conventional digital palpation and ultrasound of the cricothyroid membrane in obese women in labour. Anaesthesia.

[43] Wang B., Wang M., Yang F., Zheng C., Yu T., Xu J., Chen Y., Yao W. (2022). Predicting difficult intubation: The hyomental distance ultrasound evaluation is superior to the thyromental distance. Anaesth. Crit. Care Pain Med..

[44] Ganjoo S., Dhamija E., Garg R., Bhatnagar S., Mishra S., Bharati S.J., Gupta N., Kumar V. (2025). To assess the impact of advanced airway imaging (3D CT reconstruction and virtual endoscopy of airway) on airway management in adult patients undergoing head and neck cancer surgeries under general anaesthesia: Randomised controlled study (3D-ARC Study: 3D Airway Reconstruction for Cancer airway management). Indian J. Anaesth..

[45] Zhou Y., Han Y., Li Z., Zhao Y., Yang N., Liu T., Li M., Wang J., Guo X., Xu M. (2021). Preoperative X-ray CCAR is applicable for prediction of difficult laryngoscopy in patients with cervical spondylosis. BMC Anesthesiol..

[46] De Rosa S., Bignami E., Bellini V., Battaglini D. (2025). The Future of Artificial Intelligence Using Images and Clinical Assessment for Difficult Airway Management. Anesth. Analg..

[47] Kim J.H., Jung H.S., Lee S.E., Hou J.U., Kwon Y.S. (2024). Improving difficult direct laryngoscopy prediction using deep learning and minimal image analysis: A single-center prospective study. Sci. Rep..

[48] Wu J., Yao Y., Zhang G., Li X., Peng B. (2024). Difficult Airway Assessment Based on Multi-View Metric Learning. Bioengineering.

[49] Wünsch V.A., Bommes H., Germer S., Piltan M., Breitfeld P., Latus S., Krause L., Zöllner C., Gundler C., Petzoldt M. (2026). Pre-operative three-dimensional face scans for predicting difficult facemask ventilation: A prospective development study. Anaesthesia.

[50] Zhou C.M., Wang Y., Xue Q., Yang J.J., Zhu Y. (2022). Predicting difficult airway intubation in thyroid surgery using multiple machine learning and deep learning algorithms. Front. Public Health.

[51] Naik N.B., Mathew P.J., Kundra P. (2024). Scope of artificial intelligence in airway management. Indian J. Anaesth..

[52] Li S., Huang J., Xiao Z., Fan C. (2025). Advances in Machine Learning Prediction Models for the Screening of Obstructive Sleep Apnea in Adults. Nat. Sci. Sleep.

[53] Madadi F., Dabbagh A., Sabouri A.S. (2025). Artificial Intelligence in Pediatric Anesthesia. Anesthesiol. Clin..

[54] Hansel J., Rogers A.M., Lewis S.R., Cook T.M., Smith A.F. (2022). Videolaryngoscopy versus direct laryngoscopy for adults undergoing tracheal intubation. Cochrane Database Syst. Rev..

[55] Karamchandani K., Wheelwright J., Yang A.L., Westphal N.D., Khanna A.K., Myatra S.N. (2021). Emergency Airway Management Outside the Operating Room: Current Evidence and Management Strategies. Anesth. Analg..

[56] Sorbello M., Paternò D.S., Lo Giudice E.C., Maniaci A., Pappalardo F., La Via L. (2026). Learning curves and demographic influence on skill acquisition of artificial intelligence-assisted videolaryngoscopy training with the LarynGuide system: A pilot simulation study. Anaesth. Rep..

[57] McKechnie A., Iliff H.A., Black R., Ahmad I., Chesworth A., Chesworth P., Davis N., Griffiths C. (2025). Airway management in patients living with obesity: Best practice recommendations from the Society for Obesity and Bariatric Anaesthesia: Endorsed by the All Wales Airway Group, Scottish Airway Group and Difficult Airway Society. Anaesthesia.

[58] Seet E., Nagappa M., Wong D.T. (2021). Airway Management in Surgical Patients With Obstructive Sleep Apnea. Anesth. Analg..

[59] Oliveira J.T.M., Medeiros H., Andrino W. (2024). Prevalence of obstructive sleep apnea syndrome and predictors of difficult orotracheal intubation. Rev. Assoc. Medica Bras. (1992).

[60] Arora L., Sharma S., Carillo J.F. (2024). Obesity and anesthesia. Curr. Opin. Anaesthesiol..

[61] Wojcikiewicz T., Cousins J., Margarson M. (2018). The bariatric airway. Br. J. Hosp. Med..

[62] Richa F., El-Hage C., Chalhoub V., Yazbeck P. (2023). Prediction of difficult laryngoscopy and/or intubation among morbidly obese patients: Upper lip bite test versus modified Mallampati classification. J. Perioper. Pract..

[63] Kheirabadi D., Honarmand A., Rasouli M.R., Safavi M.R., Maracy M.R. (2021). Comparison of airway assessment tests for prediction of difficult intubation in obese patients: Importance of thyromental height and upper lip bite test. Minerva Anestesiol..

[64] Bellizzi M.G., Pace A., Iannella G., Maniaci A., Paternò D.S., Tutino S., Sorbello M., Ronsivalle S.M., Magliulo G., Greco A. (2025). Airway Management in Obstructive Sleep Apnea: A Comprehensive Review of Assessment Strategies, Techniques, and Technological Advances. Healthcare.

[65] Mushambi M.C., Athanassoglou V., Kinsella S.M. (2020). Anticipated difficult airway during obstetric general anaesthesia: Narrative literature review and management recommendations. Anaesthesia.

[66] Prior C.H., Burlinson C.E.G., Chau A. (2022). Emergencies in obstetric anaesthesia: A narrative review. Anaesthesia.

[67] O’Sullivan É., Tan T. (2025). Obstetric Airway Challenges. Anesthesiol. Clin..

[68] Šklebar I., Habek D., Berić S., Goranović T. (2023). Airway Management Guidelines in Obstetrics. Acta Clin. Croat..

[69] Lee A., Estevez M.G., Le Gouez A., Mercier F.J. (2024). Cesarean delivery: Clinical updates. Best Pract. Res. Clin. Anaesthesiol..

[70] Mossinelli C., Pietrobon G., Zorzi S., Tagliabue M., Chu F., Tomarchio E., Sances D., Venturino M., Ansarin M. (2025). Airway assessment and management in head and neck cancer surgery. Acta Otorhinolaryngol. Ital..

[71] Jain D., Khan Joad A.S. (2020). Head and neck radiotherapy—A risk factor for anaesthesia?. Indian J. Anaesth..

[72] Başpınar Ş.M., Günüşen İ., Sergin D., Sargın A., Balcıoğlu S.T. (2022). Evaluation of anthropometric measurements and clinical tests in the diagnosis of difficult airway in patients undergoing head and neck surgery. Turk. J. Med. Sci..

[73] Gupta R., Gupta N., Kumar V., Garg R., Bharati S.J., Mishra S., Bhatnagar S. (2021). El-Ganzouri multivariate risk index based airway management in head and neck cancer patients: A retrospective analysis of 1000 patients in a tertiary care center. J. Anaesthesiol. Clin. Pharmacol..

[74] De Cassai A., Boscolo A., Rose K., Carron M., Navalesi P. (2020). Predictive parameters of difficult intubation in thyroid surgery: A meta-analysis. Minerva Anestesiol..

[75] Falcetta S., Ahmad I., Paternò D.S., Sorbello M. (2025). Anesthesia management in thyroid surgery. Curr. Opin. Anaesthesiol..

[76] McCauley P., Moore M., Duggan E. (2022). Anaesthesia for reconstructive free flap surgery for head and neck cancer. Br. J. Hosp. Med..

[77] McNarry A.F., Ward P.A., Paternò D.S., Sorbello M. (2025). Oxygenation techniques in head and neck surgery. Curr. Opin. Anaesthesiol..

[78] Kornas R.L., Owyang C.G., Sakles J.C., Foley L.J., Mosier J.M. (2021). Evaluation and Management of the Physiologically Difficult Airway: Consensus Recommendations From Society for Airway Management. Anesth. Analg..

[79] Karamchandani K., Nasa P., Jarzebowski M., Brewster D.J., De Jong A., Bauer P.R., Berkow L., Brown C.A., Cabrini L., Casey J. (2024). Tracheal intubation in critically ill adults with a physiologically difficult airway. An international Delphi study. Intensive Care Med..

[80] Myatra S.N., Divatia J.V., Brewster D.J. (2022). The physiologically difficult airway: An emerging concept. Curr. Opin. Anaesthesiol..

[81] Russotto V., Tassistro E., Myatra S.N., Parotto M., Antolini L., Bauer P., Lascarrou J.B., Szułdrzyński K., Camporota L., Putensen C. (2022). Peri-intubation Cardiovascular Collapse in Patients Who Are Critically Ill: Insights from the INTUBE Study. Am. J. Respir. Crit. Care Med..

[82] Gibbs K.W., Semler M.W., Driver B.E., Seitz K.P., Stempek S.B., Taylor C., Resnick-Ault D., White H.D., Gandotra S., Doerschug K.C. (2024). Noninvasive Ventilation for Preoxygenation during Emergency Intubation. N. Engl. J. Med..

[83] Pitre T., Liu W., Zeraatkar D., Casey J.D., Dionne J.C., Gibbs K.W., Ginde A.A., Needham-Nethercott N., Rice T.W., Semler M.W. (2025). Preoxygenation strategies for intubation of patients who are critically ill: A systematic review and network meta-analysis of randomised trials. Lancet Respir. Med..

[84] La Via L., Cuttone G., Senussi Testa T., Duarte-Medrano G., Nuno-Lambarri N., Deana C., Maniaci A., Paternò D.S., Zdravkovic I., Sorbello M. (2025). Non-Invasive Positive Pressure Ventilation for Pre-Oxygenation of Critically Ill Patients Before Intubation. J. Clin. Med..

[85] Sorbello M., Paternò D.S., Zdravkovic I., La Via L. (2025). Pharmacological approach to rapid sequence induction/intubation: A contemporary perspective. Curr. Opin. Anaesthesiol..

[86] DeMasi S.C., Casey J.D., Semler M.W. (2025). Evidence-based Emergency Tracheal Intubation. Am. J. Respir. Crit. Care Med..

[87] De Jong A., Myatra S.N., Roca O., Jaber S. (2022). How to improve intubation in the intensive care unit. Update on knowledge and devices. Intensive Care Med..

[88] Long B., Gottlieb M. (2024). Emergency medicine updates: Endotracheal intubation. Am. J. Emerg. Med..

[89] Fiadjoe J.E., Nishisaki A., Jagannathan N., Hunyady A.I., Greenberg R.S., Reynolds P.I., Matuszczak M.E., Rehman M.A., Polaner D.M., Szmuk P. (2016). Airway management complications in children with difficult tracheal intubation from the Pediatric Difficult Intubation (PeDI) registry: A prospective cohort analysis. Lancet Respir. Med..

[90] Sequera-Ramos L., Laverriere E.K., Garcia-Marcinkiewicz A.G., Zhang B., Kovatsis P.G., Fiadjoe J.E. (2022). Sedation versus General Anesthesia for Tracheal Intubation in Children with Difficult Airways: A Cohort Study from the Pediatric Difficult Intubation Registry. Anesthesiology.

[91] Disma N., Asai T., Cools E., Cronin A., Engelhardt T., Fiadjoe J., Fuchs A., Garcia-Marcinkiewicz A., Habre W., Heath C. (2023). Airway management in neonates and infants: European Society of Anaesthesiology and Intensive Care and British Journal of Anaesthesia joint guidelines. Eur. J. Anaesthesiol..

[92] Zimmermann L., Maiellare F., Veyckemans F., Fuchs A., Scquizzato T., Riva T., Disma N. (2024). Airway management in pediatrics: Improving safety. J. Anesth..

[93] Geraghty L.E., Dunne E.A., Ní Chathasaigh C.M., Vellinga A., Adams N.C., O’Currain E.M., McCarthy L.K., O’Donnell C.P.F. (2024). Video versus Direct Laryngoscopy for Urgent Intubation of Newborn Infants. N. Engl. J. Med..

[94] Paternò D.S., La Via L., Lo Giudice E.C., Sorbello M. (2026). Pharmacological approach to awake tracheal intubation. Curr. Opin. Anaesthesiol..

[95] Cox L., Tebbett A. (2022). Videolaryngoscopy versus direct laryngoscopy for endotracheal intubation of cardiac arrest patients in hospital: A systematic literature review. Resusc. Plus.

[96] Di Giacinto I., Sorbello M., Falcetta S., Esposito C., Cortese G., Cataldo R., Serio P., Battaglini D. (2026). Ten tips for a safe approach in airway management. J. Anesth. Analg. Crit. Care.

[97] Huang J., Galal G., Etemadi M., Vaidyanathan M. (2022). Evaluation and Mitigation of Racial Bias in Clinical Machine Learning Models: Scoping Review. JMIR Med. Inform..

[98] Banerjee I., Bhattacharjee K., Burns J.L., Trivedi H., Purkayastha S., Seyyed-Kalantari L., Patel B.N., Shiradkar R., Gichoya J. (2023). “Shortcuts” Causing Bias in Radiology Artificial Intelligence: Causes, Evaluation, and Mitigation. J. Am. Coll. Radiol..

[99] Khor S., Haupt E.C., Hahn E.E., Lyons L.J.L., Shankaran V., Bansal A. (2023). Racial and Ethnic Bias in Risk Prediction Models for Colorectal Cancer Recurrence When Race and Ethnicity Are Omitted as Predictors. JAMA Netw. Open.

[100] Yang J., Soltan A.A.S., Eyre D.W., Clifton D.A. (2023). Algorithmic fairness and bias mitigation for clinical machine learning with deep reinforcement learning. Nat. Mach. Intell..

[101] Li F., Wu P., Ong H.H., Peterson J.F., Wei W.Q., Zhao J. (2023). Evaluating and mitigating bias in machine learning models for cardiovascular disease prediction. J. Biomed. Inform..

[102] Sahi F., Mather H., Murchan P., Lanzinger M. (2025). We Need to Stay Flexible: Making a Case for Fiberoptic Intubation Skills. Cureus.

[103] Davis S.E., Dorn C., Park D.J., Matheny M.E. (2025). Emerging algorithmic bias: Fairness drift as the next dimension of model maintenance and sustainability. J. Am. Med. Inform. Assoc..

[104] Orrock J.L., Ward P.A., McNarry A.F. (2024). Routine Use of Videolaryngoscopy in Airway Management. Int. Anesthesiol. Clin..

[105] Parks A., Law J.A., Kovacs G. (2025). Wake up!: A novel, cadaver-based approach to training emergency physicians in awake intubation. Can. J. Emerg. Med..

