# Supplementary material for: Beyond Mallampati: From Universal Bedside Tests to Precision Airway Risk Stratification
Source: J Pers Med. 2026 Oct 16;16(10):532. doi: 10.3390/jpm16100532 (PMC13706562; doi:10.3390/jpm16100532)
Supplement: Supplementary file 1 [file jpm-16-00532-s001.zip › jpm-4545538-supplementary.pdf]

Supplementary Material S1 — Search strategy

Manuscript: Beyond Mallampati: From Universal Bedside Tests to Precision Airway Risk Stratification. A Narrative Review

S1.1 Databases and dates

PubMed/MEDLINE was searched from inception to 31 July 2026. The search was supplemented by hand-searching the reference lists of all retrieved systematic reviews, meta-analyses and society guidelines, and by forward citation checking of the two most recent guideline documents (ESAIC 2025; DAS 2025).

S1.2 Search blocks

Searches were constructed by combining a population/procedure block with one domain block at a time. Free-text terms were combined with MeSH headings where available.

Block A — airway management and difficulty (used in every search)

```
("airway management"[MeSH] OR "airway management" OR "difficult airway" OR "difficult intubation"
OR "difficult laryngoscopy" OR "difficult mask ventilation" OR "difficult facemask ventilation" OR
"intubation, intratracheal"[MeSH] OR "tracheal intubation")
```

Block B — bedside predictive tests

```
(Mallampati OR "thyromental distance" OR "thyromental height" OR "upper lip bite test" OR "mouth
opening" OR "interincisor" OR "sternomental" OR "neck circumference" OR "Wilson score" OR "El-
Ganzouri" OR "Simplified Airway Risk Index" OR MACOCHA) AND ("diagnostic accuracy" OR sensitivity
OR specificity OR "predictive value" OR "likelihood ratio" OR reproducibility OR "observer
variation"[MeSH])
```

Block C — point-of-care ultrasound

```
(ultrasound OR ultrasonography OR sonograph* OR "point-of-care ultrasound" OR POCUS) AND ("pre-
epiglottic" OR "skin to epiglottis" OR "skin to hyoid" OR "skin to vocal cords" OR "hyomental
distance ratio" OR "tongue thickness" OR "cricothyroid membrane")
```

Block D — imaging

```
("computed tomography" OR "magnetic resonance" OR cephalometr* OR "three-dimensional" OR "virtual
endoscopy" OR "3D reconstruction" OR radiograph*)
```

Block E — machine learning and artificial intelligence

```
("machine learning"[MeSH] OR "artificial intelligence"[MeSH] OR "deep learning" OR "neural
network*" OR "facial recognition" OR "face scan" OR "voice analysis" OR "predictive model*" OR
"digital phenotyping")
```

Block F — populations (each run separately against Block A)

```
obesity[MeSH] OR obese OR bariatric OR "obstructive sleep apnea"[MeSH] OR OSA

pregnancy[MeSH] OR obstetric* OR caesarean OR cesarean OR parturient

"head and neck neoplasms"[MeSH] OR "head and neck cancer" OR radiotherapy[MeSH] OR thyroidectomy
OR "thyroid surgery"

"critical illness"[MeSH] OR "critically ill" OR "intensive care" OR "emergency department" OR
"physiologically difficult airway" OR preoxygenation

child[MeSH] OR paediatric OR pediatric OR neonate* OR infant[MeSH] OR syndromic
```

Block G — guidelines, equity and methodology

```
(guideline[Publication Type] OR "practice guideline"[Publication Type] OR consensus OR Delphi)

("algorithmic bias" OR fairness OR "racial bias" OR "skin tone" OR "external validation" OR
calibration)
```

S1.3 Selection criteria

Included: systematic reviews and meta-analyses of diagnostic accuracy; society guidelines and consensus statements; large prospective cohorts and registries; randomised controlled trials; and primary observational studies where these illustrated a parameter, a population or a methodological limitation not otherwise represented in the retrieved syntheses.

Excluded: case reports and small case series (fewer than five cases); studies not available in English; conference abstracts without full publication; and articles for which the outcome definition could not be determined.

Prioritisation: where several sources supported the same statement, the most recent and methodologically strongest was cited, with preference for systematic reviews and meta-analyses over primary studies, and for externally validated over development-only prediction models.

S1.4 Use of comparative terms

Comparative expressions used in the manuscript ("best-supported", "highest AUROC", "most extensively studied") refer to the body of evidence retrieved by the above searches and identified in Tables 1 and 2, and are not the product of a formal ranking or network meta-analysis. Where estimates originate from different evidence syntheses, this is stated in the text and in the relevant table or figure legend.

S1.5 Limitations of the search

The search was conducted in a single bibliographic database, supplemented by hand-searching. Because this is a narrative rather than a systematic review, no dual independent screening, formal risk-of-bias assessment or quantitative synthesis was undertaken, and study selection reflects the authors' judgement of representativeness. The manuscript was prepared with attention to the six domains of the SANRA scale for narrative reviews.
